# Systemic treatment with liver X receptor agonists raises apolipoprotein E, cholesterol, and amyloid-β peptides in the cerebral spinal fluid of rats

**DOI:** 10.1186/1750-1326-5-44

**Published:** 2010-10-29

**Authors:** Sokreine Suon, Jie Zhao, Stephanie A Villarreal, Nikesh Anumula, Mali Liu, Linda M Carangia, John J Renger, Celina V Zerbinatti

**Affiliations:** 1 Department of Neurosymptomatic Disorders, Merck Research Laboratories, West Point, PA 19486, USA; 2 Bioanalytics and Pathology, Merck Research Laboratories, West Point, PA 19486, USA; 3 Laboratory Animal Resources, Merck Research Laboratories, West Point, PA 19486, USA

## Abstract

**Background:**

Apolipoprotein E (apoE) is a major cholesterol transport protein found in association with brain amyloid from Alzheimer's disease (AD) patients and the *ε4 *allele of apoE is a genetic risk factor for AD. Previous studies have shown that apoE forms a stable complex with amyloid β (Aβ) peptides *in vitro *and that the state of apoE lipidation influences the fate of brain Aβ, i.e., lipid poor apoE promotes Aβ aggregation/deposition while fully lipidated apoE favors Aβ degradation/clearance. In the brain, apoE levels and apoE lipidation are regulated by the liver X receptors (LXRs).

**Results:**

We investigated the hypothesis that increased apoE levels and lipidation induced by LXR agonists facilitates Aβ efflux from the brain to the cerebral spinal fluid (CSF). We also examined if the brain expression of major apoE receptors potentially involved in apoE-mediated Aβ clearance was altered by LXR agonists. ApoE, cholesterol, Aβ40, and Aβ42 levels were all significantly elevated in the CSF of rats after only 3 days of treatment with LXR agonists. A significant reduction in soluble brain Aβ40 levels was also detected after 6 days of LXR agonist treatment.

**Conclusions:**

Our novel findings suggest that central Aβ lowering caused by LXR agonists appears to involve an apoE/cholesterol-mediated transport of Aβ to the CSF and that differences between the apoE isoforms in mediating this clearance pathway may explain why individuals carrying one or two copies of APOE *ε4 *have increased risk for AD.

## Background

Alzheimer's disease (AD) is a neurodegenerative disease characterized by the progressive loss of memory and cognitive function [1]. The presence of amyloid-β (Aβ) peptide deposits in the hippocampal and cortical regions of the brain is a major hallmark of AD pathology. Aβ peptides, mainly Aβ40 and Aβ42, are released from the transmembrane amyloid precursor protein (APP) following sequential cleavage by β- and γ-secretases and have been shown to cause toxicity to both neurons and glia cells *in vitro *and *in vivo *[1,2]. The most significant genetic association reported for late-onset AD is with apolipoprotein E (apoE), the main lipid transporter protein in the central nervous system (CNS) [3,4]. Three human apoE isoforms arise from polymorphisms within the APOE gene, named E2, E3 and E4. While only 15% of the normal population carries apoE4, up to 70% of AD patients have one or two copies of apoE4.

The mechanism by which apoE4 increases the risk for AD is not yet clear. It has been established that both healthy individuals and AD patients carrying apoE4 have increased brain amyloid burden [5,6]. Likewise, APP transgenic (APP-Tg) mice expressing human apoE4 in replacement of mouse apoE (apoE4 knock-in, KI) have increased brain amyloid load compared to apoE3 KI mice [7]. Furthermore, it has been previously shown that binding of apoE to Aβ *in vitro *is isoform- and lipid-dependent [8,9]. When associated with lipids, apoE3 binds Aβ more efficiently than apoE4. However, apoE's ability to bind Aβ is reduced and the isoform-dependent Aβ binding differences are lost when apoE is lipid-free. Therefore, due to its ability to bind Aβ, particularly the more amyloidogenic Aβ42, apoE appears to play an important role in both the aggregation and the clearance of Aβ within and out of the brain parenchyma.

In addition to increased brain amyloid load, apoE4 KI mice have reduced levels of CNS apoE and it has been postulated that the overall lower levels of apoE protein may explain the increased amyloid load and risk for AD in apoE4 carriers [7,10,11]. Astrocyte-secreted apoE4 also has decreased association with lipids when compared to apoE3, a difference that may underlie the reduced ability of apoE4 to bind Aβ and to promote its clearance [11,12]. The majority of lipids associated with astrocyte-secreted apoE are contributed by the ATP-binding cassette protein A1 (ABCA1), and both apoE and ABCA1 are liver X receptors (LXR)-target genes [13,14].

LXRα and LXRβ are nuclear hormone receptors involved in lipid metabolism and inflammatory signaling throughout the body [15]. It has been previously shown that synthetic LXR agonists regulate the expression of apoE and ABCA1 in astrocytes suggesting that LXRs play an important role in the regulation of lipid metabolism in the CNS [16]. Increased levels of brain ABCA1 and apoE observed with short-term (6-10 days) and prolonged (4 months) systemic treatment with synthetic LXR agonists was also associated with reduced brain Aβ burden in mice [17-23]. The mechanism by which LXR activation reduces brain amyloid appears to be via increased Aβ clearance since APP processing was not altered by LXR agonist compounds [22,23]. Potential clearance pathways may involve the endocytosis of Aβ/apoE-lipid complexes via apoE receptors such as the low-density lipoprotein receptor (LDLR) and the low-density lipoprotein receptor-related protein 1 (LRP1), which are highly expressed in neurons and glia and have been shown to regulate the overall levels of CNS apoE [24-27]. Other Aβ clearance pathways are through elimination at the blood-brain barrier (BBB) and the CSF [28,29]. These pathways are not well understood and may also involve Aβ/apoE-lipid particles binding to apoE receptors.

In the present study, we investigated the modulation of apoE and Aβ levels by LXR agonists in the CSF. Because this Aβ clearance pathway is likely to be saturated in APP overexpressing mice and CSF samples free of blood contamination can be difficult to obtain from mice, we used rats for these studies. We show, for the first time, that systemic administration of LXR agonists causes a robust and fast increase in apoE, cholesterol, and endogenous Aθ in the CSF of rats. Consistently, LXR agonist treatment markedly increases the levels of LXR target genes apoE and ABCA1 in cells of the choroid plexus and ependyma, suggesting that central engagement of LXRs may improve the clearance of parenchymal amyloid to the ventricular system, contributing to the overall reduction of brain amyloidosis.

## Results

**Figure 1:**
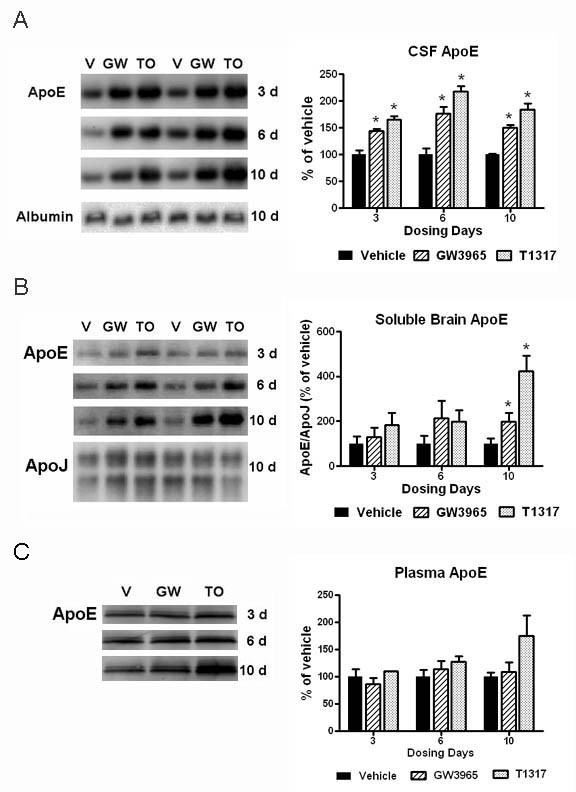
**CSF and brain apoE levels were markedly increased with LXR agonist treatment**. The levels of apoE in the CSF, soluble brain extracts, and plasma following systemic treatment with LXR agonists T1317 and GW3965 at 30 mpk for 3, 6, and 10 days were evaluated by Western blot analysis as described in Methods. A: Representative blots showing changes in apoE levels in the CSF of rats treated with vehicle or LXR agonists (left panel). Albumin was used as a loading control for all CSF blots. Densitometric analysis of Western blots for CSF apoE (right panel). B: DEA extracted soluble brain homogenates were prepared as described in Methods. Equal amounts of supernatant proteins were loaded per lane and apoJ was used as an additional loading control. Representative blots showing changes in apoE observed in the soluble brain fraction of rats treated with vehicle, T1317 and GW3965 (left panel). Densitometric analysis of Western blots for soluble brain apoE (right panel). C: Representative blots showing changes of apoE levels in the plasma of rats treated with vehicle or LXR agonists (left panel). Densitometric analysis of Western blots for plasma apoE (right panel). * p < 0.05 compared to vehicle treated controls by ANOVA, N = 4 per group.

Systemic administration of LXR agonists for 6-10 days had been previously shown to upregulate brain apoE and reduce Aβ levels in both APP transgenic and wild-type mice [17-23]. Because apoE is abundant in the CSF and CSF apoE is thought to derive entirely from within the brain, we sought to investigate the effects of LXR agonist treatment on CSF apoE and Aβ levels. A marked up-regulation of apoE levels in the CSF was detected by Western blotting analysis following systemic treatment with LXR agonists T1317 and GW3965 at 30 mpk (Figure 1A). This increase was statistically significant after 3 days of dosing and similar to that observed with 6 and 10 days of treatment. Treatment with the more potent full LXR agonist T1317 produced a larger increase in CSF apoE than the less potent partial agonist GW3965. Compound levels in the brain and plasma were also higher for T1317 than for GW3965 (data not shown), likely contributing to the differential degree of efficacy of the two compounds. No significant changes were observed in the CSF albumin levels.

Soluble apoE levels were also significantly elevated in the brain following treatment with LXR agonists. DEA-soluble extracts obtained from the right forebrain (cortex and hippocampus) were analyzed for apoE content by Western blotting (Figure 1B). Up-regulation of soluble apoE was clearly observed after 6 days of dosing, but the differences were only statistically significant after 10 days of treatment. This LXR agonist-mediated effect was specific to apoE as there were no significant changes in the soluble levels of brain apoJ, which is not directly regulated by LXRs. Consistent with the findings for the CSF, T1317 showed a more potent effect in increasing soluble apoE brain levels than GW3965. The delayed increase in brain apoE when compared to CSF apoE levels was also observed in the plasma, where elevation in apoE levels were more evident following 10 days of treatment with LXR agonists (Figure 1C). In summary, up-regulation of the LXR target gene apoE was observed in the CSF, the brain parenchyma and plasma within a few days of systemic administration with LXR agonists.

**Figure 2:**
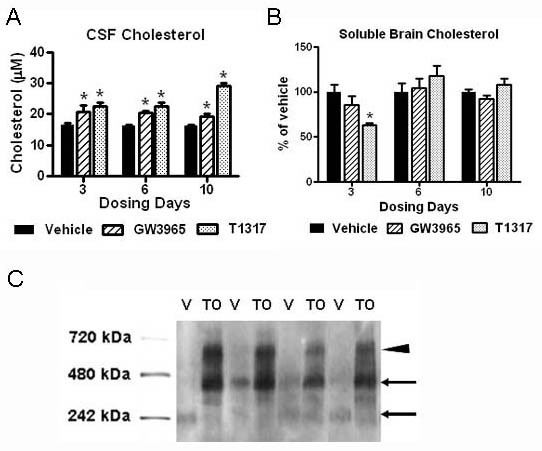
**CSF and soluble brain cholesterol were inversely altered by LXR agonist treatment**. The levels of cholesterol in CSF and soluble brain extracts were evaluated using the Amplex Red Cholesterol Kit as described in Methods. A: Cholesterol levels in the CSF of rats treated with LXR agonists in comparison to the vehicle control. B: Cholesterol levels in the DEA-soluble brain fraction of rats treated with LXR agonists in comparison to the vehicle control. * p < 0.05 compared to vehicle treated controls by ANOVA, N = 4 per group. C: CSF samples were separated in native gel as described in Methods and then blotted with an anti-apoE antibody. Representative blot showing apoE immunoreactive bands in the CSF of rats treated with vehicle or T1317 for 3 days. CSF from vehicle-treated rats shows apoE-immunoreactive bands around 240 and 400 kD (←), while CSF from T1317-treated animals had strongest apoE-immunoreactivity in bands detected at approximately 400 and 650 kD (◀).

Because LXR agonists have also been shown to upregulate the cholesterol efflux pump ABCA1 in the CNS, we evaluated total cholesterol levels in CSF samples from rats treated with LXR agonists in comparison to the vehicle control (Figure 2A). CSF cholesterol was significantly increased by LXR agonists at all time points examined, with the greatest change observed after 10 days of dosing with T1317 (2-fold increase). Interestingly, we observed a concomitant decrease of cholesterol in the DEA-soluble brain fraction of rats treated with T1317 after 3 days of dosing, which was reversed to normal levels with extended treatment (Figure 2B). These results are consistent with the up-regulation of cholesterol synthesis that follows increased cholesterol efflux induced by LXR agonists [30].

When CSF samples were separated in native gels, immunoreactivity associate with apoE in T1317 treated rats was markedly increased in bands at higher molecular weight than the bands present in the CSF of vehicle control rats (Figure 2C). While vehicle CSF showed faint apoE-immunoreactive bands at 240 and around 400 kD (←), CSF from T1317-treated animals had strong apoE-immunoreactivity in bands detected at approximately 400 and 650 kD (◀). These results suggested that larger apoE-lipid particles were likely present in the rat CSF following LXR agonist treatment.

**Figure 3:**
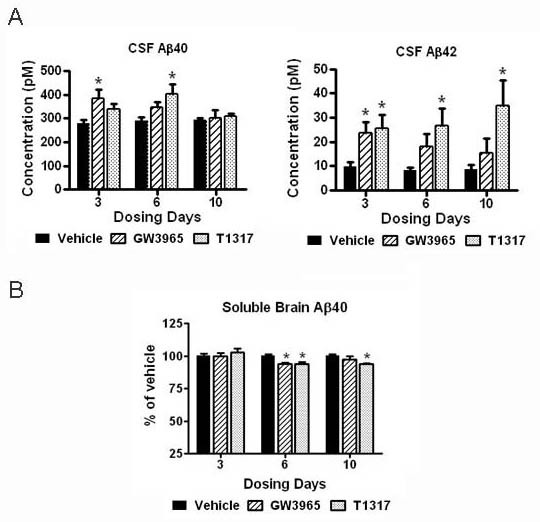
**CSF Aβ levels were increased and soluble brain Aβ levels were decreased by LXR agonists**. The levels of Aβ40 and Aβ42 in the CSF and soluble brain extracts were evaluated by ELISA as described in Methods. A: Aβ40 and Aβ42 levels in the CSF of rats treated with LXR agonists in comparison to the vehicle control. B: Aβ40 levels in the DEA-soluble brain fraction of rats treated with LXR agonists in comparison to the vehicle control. * p < 0.05 compared to vehicle treated controls by ANOVA, N = 4 per group.

We next asked if the increased transport of apoE-cholesterol to the CSF induced by LXR agonists was accompanied by increased transport of Aβ peptides and reduced brain amyloid. As shown in Figure 3A, Aβ40 and Aβ42 levels were significantly increased in the CSF of rats treated with LXR agonists for 3 days. The effect was most robust following 6 days of treatment with T1317 and coincident with a small, but significant decrease in DEA-soluble brain Aβ40 (Figure 3B). DEA-soluble brain Aβ42 was below the minimum detection level of the Aβ rodent ELISA. Taken together, these results support the hypothesis that apoE-lipid transport may influences Aβ efflux from the brain to the CSF.

**Figure 4:**
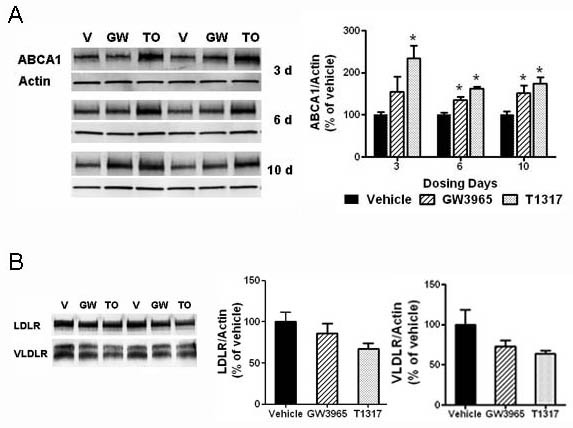
**Brain ABCA1 levels were upregulated, while LDLR and VLDLR were slightly reduced by LXR agonist treatment**. The brain levels of the LXR target gene ABCA1 and of the apoE receptors LDLR and VLDLR were evaluated in RIPA extracted brain homogenates by Western blot analysis as described in Methods. Equal amounts of supernatant proteins were loaded per lane and actin was used as an additional loading control. A: Representative blots showing ABCA1 levels in the brain of rats treated with vehicle or LXR agonists (left panel). Densitometric analysis of Western blots for ABCA1 (right panel). B: Representative blots showing LDLR and VLDLR steady-state levels in the brain of rats treated with vehicle, T1317, and GW3965 for 10 days (left panel). Densitometric analysis of Western blots for LDLR and VLDLR (right panel). * p < 0.05 compared to vehicle treated controls by ANOVA, N = 4 per group.

Brain levels of the LXR target gene ABCA1 and the apoE receptors LDLR and very low-density lipoprotein receptor (VLDLR), which may be directly or indirectly regulated by LXR, were also examined in detergent (RIPA) extracts (Figure 4). Consistent with previous published studies, ABCA1 protein levels were significantly increased by LXR agonists at all time points examined (Figure 4A). Following 10 days of compound treatment, the steady-state levels of the apoE receptors LDLR and VLDLR appeared reduced when compared to that of vehicle controls, but these differences did not reach statistical significance (Figure 4B).

**Figure 5:**
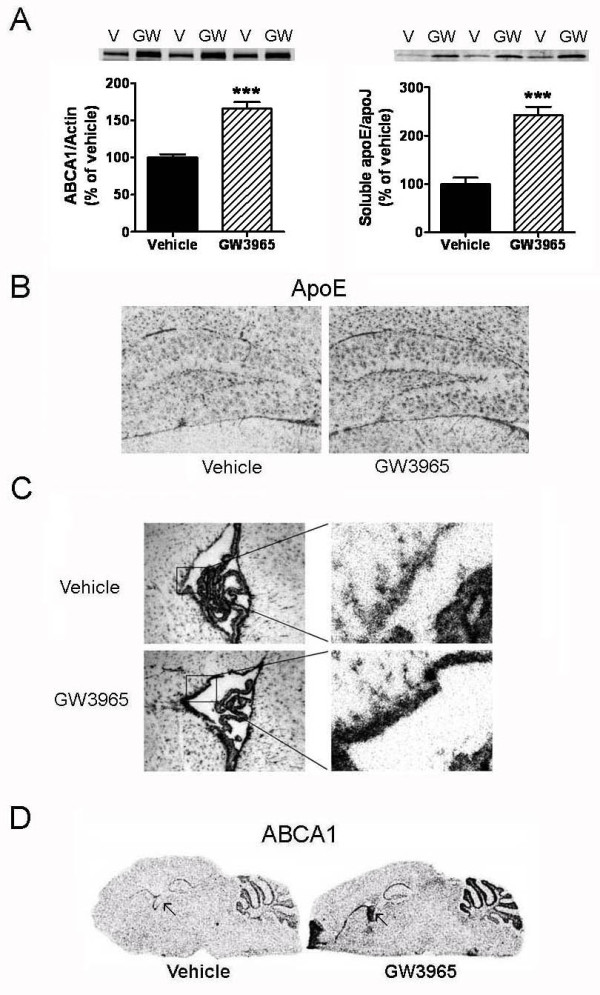
**ABCA1 and apoE mRNA expression levels were upregulated in the mouse brain by LXR agonist treatment**. ApoE and ABCA1 mRNA as well as protein expression were examined in the mouse brain by Western blot analysis and *in situ *hybridization following GW3965 treatment (50 mpk for 10 days). A: RIPA and DEA-soluble brain homogenates were prepared as described in Methods. Equal amounts of supernatant proteins were loaded per lane and actin or ApoJ was used as an additional loading control. Densitometric analysis of Western blots for ABCA1 (left panel) and apoE (right panel) levels in the brain of mice treated with vehicle or GW3965. * p < 0.05 compared to vehicle treated controls by ANOVA, N = 9 per group. B: Brain distribution of apoE mRNA by *in situ *hybridization showing predominant astrocytic expression and increased expression following treatment with GW3965. C: Marked increase of apoE mRNA was observed in the ependymal cells lining the lateral ventricle following LXR agonist treatment. D: *In situ *hybridization for ABCA1 mRNA also showing high up-regulation in the lateral ventricle by GW3965 (←).

CSF is secreted by the choroid plexus and by the ependymal cells lining the ventricles [31]. Because LXR agonist-induced changes in CSF apoE appear to occur more rapidly and at a greater degree than in the brain tissue, we examined the pattern of apoE and ABCA1 mRNA expression in the mouse brain by *in situ *hybridization following GW3965 treatment (50 mpk for 10 days). As shown in Figure 5A, both ABCA1 and soluble apoE protein levels were significantly elevated in the mouse brain, similar to the changes observed in the rat brain. By *in situ *hybridization, apoE mRNA was very abundant in the mouse brain and detected in a pattern consistent with astrocytic expression (Figure 5B). Even though apoE mRNA was noticeably upregulated throughout the brain following GW3965 treatment (Figure 5B), the ependyma region of the lateral ventricles showed a robust up-regulation of apoE mRNA by the LXR agonist (Figure 5C). ABCA1 mRNA overall distribution was consistent with both neuronal and astrocytic expression, but similar to apoE mRNA, up-regulation by GW3965 was also strong in the choroid plexus and ependyma (Figure 5D). These findings suggest that a high level of LXR activation in the choroid plexus and ependymal cells could have led to the fast and significant up-regulation of apoE and cholesterol levels observed in the rat CSF following systemic treatment with LXR agonists.

**Figure 6:**
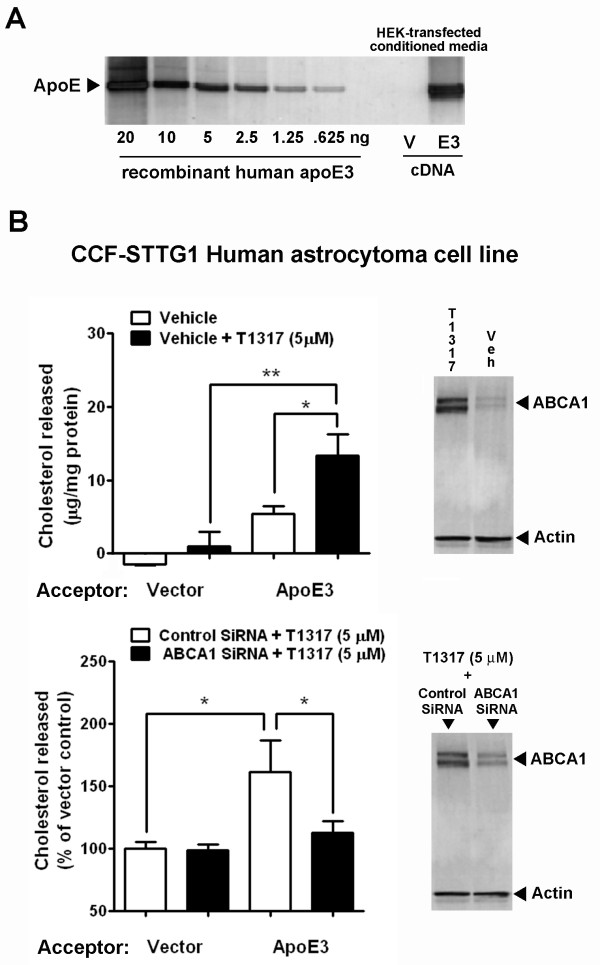
**LXR agonist-stimulated cholesterol efflux is apoE- and ABCA1- dependent**. The role of ABCA1 in the T1317-induced cholesterol efflux to exogenous apoE was examined in the CCF-STTG1 human astrocytoma cell line. A: HEK 293 cells were transiently transfected with human apoE3 cDNA (E3) or empty vector (V) and concentrated serum-free conditioned media was analysed on Western blotting for apoE content against human recombinant apoE3. B: Equal amounts of conditioned media protein containing approximately 50 μg/ml of HEK-apoE3 were added to CCF-STTG1 astrocytoma cells pre-loaded with cholesterol in serum-free media containing 5 μM T1317 or 0.05% DMSO (vehicle control). 48 h-conditioned media was then analyzed for total cholesterol and protein content while cell monolayers were lysed for Western blotting analysis of ABCA1 protein levels. Treatment with T1317 (5 μM) up-regulated the expression of ABCA1 and increased the release of cholesterol to HEK-apoE3, but not to vector-HEK media. C: Knock-down of ABCA1 with specific siRNA inhibited T1317-induced ABCA1 up-regulation and cholesterol release to HEK-apoE3 media. *p < 0.05 and **p < 0.01 by ANOVA.

Finally, to corroborate our hypothesis that LXR agonists increase apoE lipidation in the brain, we examined the ability of T1317 to increase the release of cholesterol from a brain relevant cell line to exogenously added apoE. ApoE particles were generated by transient transfection of the human apoE3 cDNA into HEK 293 cells. Conditioned serum-free media from empty vector-transfected HEK cells was used as control (Figure 6A). Cholesterol-loaded CCF-STTG1 astrocytoma cells were treated with approximately 50 μg/ml of apoE3-concentrated media or the equivalent of total protein from vector-concentrated media/ml (no detectable apoE) plus 5 μM T1317 or vehicle, and incubated in serum-free media for 48 hours. As shown in Figure 6B, total cholesterol/protein measured in the media following incubation with T1317 was significantly greater in the apoE3 media than in the vector media. In addition, T1317 did not stimulate the secretion of cholesterol to the vector media, suggesting that apoE is the putative acceptor for the released cholesterol. As expected, Western blotting of CCF-STTG1 cell lysates showed marked up-regulation of ABCA1 with T1317 treatment. To further confirm the role of ABCA1 in lipidating exogenously added apoE3, CCF-STTG1 cells were transfected with siRNA to ABCA1 (non-targeting siRNA was used as control) in the presence of 5 μM T1317. We found that precluding the up-regulation of ABCA1 by T1317 with a specific siRNA also prevented the increase in cholesterol secreted to the apoE3 media, but it had no effect in the vector media (Figure 6C). Together our results suggest that, via up-regulation of ABCA1, LXR agonists can increase the lipidation of apoE, which may improve its ability to bind and transport Aβ out of the brain.

## Discussion

Genetic and biochemical evidence suggest that apoE plays a crucial role in the pathology of AD [3,32-34]. In the CNS, apoE is the major component of HDL-like particles that deliver lipids to neurons via uptake by apoE receptors such as LRP1 and LDLR. ApoE and the cholesterol efflux pump ABCA1 are LXR-target genes and LXRs are highly expressed in glia cells, which are the primary site of apoE and lipid synthesis in the brain. Both short- and long-term administration of synthetic LXR agonists have been shown to upregulate apoE and ABCA1 levels and to reduce brain amyloid load [17-23]. We have shown here for the first time that systemic administration of LXR agonists also leads to a marked and rapid up-regulation of apoE, cholesterol, and Aβ peptides in the CSF of rats, suggesting that this pathway can contribute significantly to the elimination of Aβ from the brain.

In addition to providing cholesterol needed for synaptic plasticity and membrane maintenance, apoE is involved in the elimination of excess cholesterol from the brain and this pathway is upregulated in the neurodegenerative state [35]. In agreement with this important apoE role, administration of LXR agonist T1317 increased brain cholesterol excretion by almost 3-fold and slowed neurodegeneration in Niemann-Pick type C (NPC1) mutant mice [36]. Deletion of LXRs in mice results in several brain abnormalities, most notably the lateral ventricles are closed, lined with lipid-laden cells, and deprived of CSF content by 12 months of age [37]. Deletion of LXRs also significantly increases brain amyloid burden in APP-Tg mice [38]. Taken together, these studies implicate LXRs in the clearance of excess cholesterol and Aβ from the brain, likely involving transport to the CSF.

Amyloid-binding apoE and apoAI are the most abundant apolipoproteins in the CSF [39]. However, while CSF apoAI is derived from the plasma, CSF apoE is believed to originate entirely within the CNS [40]. CSF is produced by the choroid plexus, the ependymal cells lining the ventricles, and the brain parenchyma [41]. Besides offering support and cushion to elements of the CNS, the CSF also provides an effective pathway for elimination of metabolites from the brain via drainage to the vascular system [31]. In young and adult rats, CSF is secreted at a rate of approximately 1.3 μl/min and is replaced in its total volume about 10 times per day [42]. In humans, the total CSF volume is approximately 130 ml and is completely replaced about four times per day [31]. CSF secretion rate decreases with aging, but the overall CSF volume increases in older individuals due to a reduced rate of turnover [42,43]. These age-related changes may translate into reduced Aβ elimination via changes in CSF fluid dynamics. We found that apoE and ABCA1 mRNAs were highly upregulated by LXR agonists in the ventricular region of the mouse brain. In support of our findings, it has been previously shown that treatment with the endogenous LXR ligand 24*S*-hydroxycholesterol led to a marked increase in ABCA1 levels and cholesterol release from the apical side of the rat choroid plexus epithelial cell line TR-CSFB3 [44]. Furthermore, LXR-mediated increase in cholesterol release was greater when apoE3 instead of apoE4 was used as an acceptor [44]. Taken together with these previous published findings, our results suggest that LXRs play an important role in controlling the excretion of lipid-associated apoE into the CSF and that this mechanism may underlie changes in the ability of the CNS to control amyloid clearance via lipid particle transport out of the brain parenchyma.

The steady-state levels of brain ABCA1 were also rapidly increased with LXR agonist treatment in rats, consistently with the overall changes in apoE and cholesterol seen in the CSF. The changes induced by GW3965 treatment for ABCA1 in the mouse brain were of similar magnitude to that observed in GW3965-treated rats, and about 1.5-fold greater than the levels observed in vehicle treated controls. Correspondingly with LXR agonist treatment, a 6-fold genetic overexpression of ABCA1 in the brain led to a striking decrease of amyloid burden in APP-Tg mice [45]. Overexpression of ABCA1 was further associated with increased lipidation of astrocyte-secreted apoE, but overall reduced levels of brain apoE, suggesting that highly lipidated apoE particles have an improved clearance rate from the brain parenchyma. In contrast, ABCA1 deletion from APP-Tg mouse lines decreased apoE secretion by astrocytes and brain apoE levels with concomitant increase in amyloid deposition [46-48], further implicating apoE and lipid transport as an important route for amyloid clearance in the CNS.

Upon T1317 treatment, the CCF-STTG1 human astrocytoma cell line released cholesterol to exogenously added HEK-apoE3 conditioned media but not to HEK-control vector conditioned media suggesting that apoE is a putative acceptor for ABCA1-mediated cholesterol efflux stimulated by LXR agonists. In addition, the knock-down of ABCA1 prevented cholesterol release to HEK-apoE in the presence of T1317. These results suggest that ABCA1 up-regulation by LXR agonists leading to increased lipidation of brain apoE could modulate apoE's ability to interact with Aβ as well as its catabolism and transport within the CNS.

**Figure 7:**
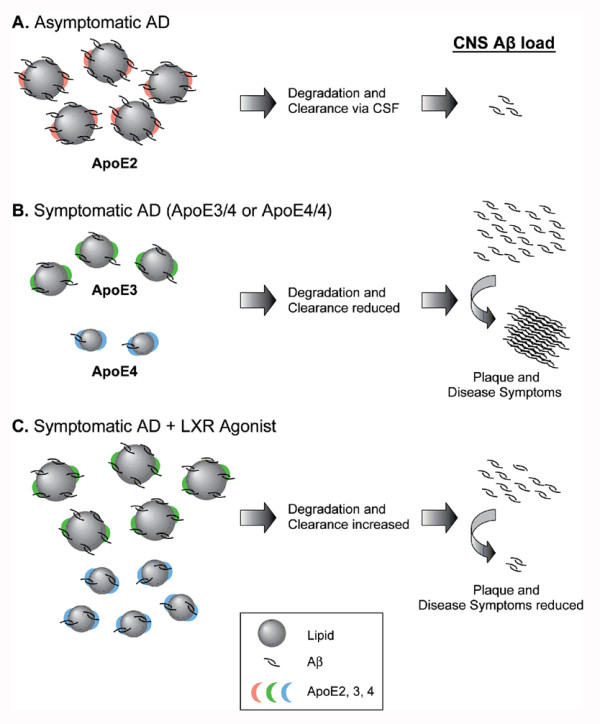
**Proposed mechanism by which LXR agonists improve apoE-mediated Aβ clearance from the brain**. Based on the differential ability of the apoE isoforms to associate with lipids, Aβ, and LDLR, the model predicts that the "protective" apoE2, which has poor binding to LDLR and therefore increased association with lipids and ability to bind Aβ, favors elimination of amyloid to the CSF reducing amyloid deposition within the brain parenchyma (A). In contrast, apoE4, which has decreased association with lipids and reduced Aβ binding capacity when compared to apoE3 and apoE2, can lead to impaired Aβ clearance, increased Aβ oligomerization and deposition into amyloid plaques (B). By raising the levels and the lipidation of apoE and potentially decreasing apoE receptors within the brain, LXR agonists favor the transport of apoE and amyloid to the CSF with subsequent decrease in brain amyloid burden, similar to the "apoE2 effect" (C).

ApoE is found in association with amyloid extracted from AD brains and *in vitro*, apoE forms a SDS-stable complex with Aβ in an apoE isoform dependent-manner [8,9,32]. This differential binding of apoE isoforms to Aβ appears to correlate with their ability to associate with lipids, with apoE4 showing decreased association with lipids and reduced ability to bind Aβ when compared to apoE3 and apoE2 [8,9]. The AD-protective apoE2 isoform, which has reduced binding to LDLR and increased association with lipids compared to apoE3, can potentially improve the transport of Aβ out of the brain, likely via the CSF [49] (Figure 7A). Because apoE4 appears to have increased clearance by apoE receptors, we hypothesize that the diminished ability of apoE4 to bind Aβ and its faster clearance rate within the brain parenchyma leads to impaired clearance of soluble Aβ, which results in an increased rate of oligomerization and deposition into neuritic plaques (Figure 7B). The reduced capacity of apoE4 to transport lipids can also lead to increased neuronal susceptibility to toxic Aβ species [50]. This model is corroborated by previous findings showing decreased CSF apoE in apoE4-KI mice, while human apoE2 expressing mice have increased apoE levels in the CSF when compared to apoE3 [51]. Furthermore, deletion of LDLR in these mice normalized the level of CSF apoE in the *ε3 *and *ε4 *genotypes to the levels observed in the *ε2 *genotype, a finding that supports the *in vitro *evidence of a faster clearance rate for apoE4 by LDLR within the brain [11]. By increasing the levels and lipidation of apoE as well as decreasing LDLR levels within the brain, LXR agonists would favor the transport of apoE and amyloid to the CSF subsequently reducing brain amyloid burden, similarly to an "apoE2 effect" (Figure 7C).

It has been previously proposed that the primary mechanism by which LXR agonists decrease Aβ burden is via increased degradation of apoE-lipid by microglia [22,23]. This mechanism may involve apoE receptors that are highly expressed in microglia, particularly LDLR. In agreement with the ability of apoE-lipid particles to bind Aβ and promote its clearance, overexpression of LDLR reduced brain apoE levels and Aβ deposition in APP Tg mice [27]. However, LDLR deletion did not result in increased amyloid deposition in the same APP Tg model [51]. Unchanged levels of amyloid could have resulted from increased transport of apoE-bound Aβ out of the brain in the absence of LDLR. Nonetheless, due to the high levels of Aβ production in APP-Tg mice, this transport mechanism was likely saturated, which prevented an overall decrease in the brain Aβ burden from occurring.

Even though LDLR is not directly regulated by LXRs, recent findings implicated the LXR-responsive ubiquitin-ligase Idol in the degradation of LDLR and VLDLR in peripheral tissues other than the liver [52,53]. Although it did not reach statistical significance, a down-regulation of LDLR and VLDLR levels was observed in the brain of rats treated with LXR agonists for 10 days, in agreement with the Idol mechanism previously described for other tissues. Taken together with these recent findings, our results suggest that LXR agonists not only increased apoE synthesis and lipidation, but also led to reduced levels of apoE receptors in the brain, which could result in further improvement of apoE-bound amyloid clearance from the CNS. This regulatory mechanism is consistent with the pivotal role of LXRs in promoting reverse cholesterol transport from tissues to the liver for excretion.

Our novel findings suggest that LXRs play an important role in the elimination of excess brain cholesterol via secretion of apoE-lipid particles into the CSF. This transport mechanism is also likely to be involved in the elimination of amyloid, particularly of Aβ42. It has been recently shown that low CSF Aβ42 levels are more closely linked to the presence of the *ε4 *allele of APOE than to clinical status of AD patients, which suggests that Aβ42 levels in the human CSF are highly influenced by apoE genotype [54,55]. The APOE4 *ε4 *genotype is associated with increased brain amyloid load in AD patients and normal individuals, as well as in APP Tg mice [55,56]. Recent studies have also shown that apoE4 KI mice have decreased steady-state levels of apoE protein in the CNS when compared to apoE3 KI mice [7,11]. Therefore, reduced levels of Aβ42 in the CSF and increased brain amyloid load in apoE4 carriers could be explained in part by the reduced capacity of apoE4 to bind and transport amyloid out of the brain.

## Conclusions

In summary, our original findings provide new insights into the function of LXRs in brain cholesterol homeostasis and suggest that the CSF is likely a major pathway for elimination of CNS-originated apoE-lipid particles and Aβ. Our results also provide further evidence to the hypothesis that the increased risk for AD associated with apoE4 is related to its reduced ability to associate with lipids and therefore to bind and remove toxic Aβ42 from the CNS, supporting the rationale for the development of safe LXR agonists for the treatment and prevention of AD.

## Methods

### Animals

Sprague Dawley male rats (~1 month of age) were dosed subcutaneously with vehicle (propylene glycol) or with synthetic LXR agonists at 30 mg/Kg (mpk) daily for 3, 6 or 10 days. T0901317 was purchased from Cayman Chemical and GW3965 was synthesized as previously described [57]. CSF, plasma, and brain were collected from non-fasted animals approximately 18 hours after last dose. C57Bl6/SJL male mice (6 months of age) were dosed subcutaneously with vehicle (propylene glycol) or GW3965 at 50 mpk for 10 days. Brains were harvested from non-fasted animals approximately 18 hours after last dose following transcardial perfusion with ice-cold phosphate buffer saline (PBS) containing 3 U/ml of heparin. All animals were handled according to the Public Health Service (PHS) Policy on Humane Care and Use of Laboratory Animals guidelines and the study protocol was approved by the Institutional Animal Care and Use Committee (IACUC).

### Western blotting

Rat forebrain samples including the cortex and hippocampus from the right hemisphere were homogenized in 4X volume of RIPA buffer (Sigma-Aldrich) containing complete protease inhibitor cocktail (Roche) and 1 mM phenylmethylsulfonyl fluoride (PMSF), followed by incubation with RIPA buffer for 30 additional minutes at 4°C. One ml volume of each sample was spun at 14,000 rpm for 15 minutes at 4°C. Protein concentration was measured in the supernatant using a BCA assay kit (BioRad). Equal amounts of sample protein in Laemmli buffer were separated on 4-15% Tris-HCl polyacrylamide SDS gels (BioRad) and transferred to polyvinylidene fluoride (PVDF) membranes. Blots were placed in blocking solution with 10% non-fat milk in phosphate buffer saline with 0.05% Tween-20 (PBS-T) for 1 h, followed by incubation with various primary antibodies diluted in 5% nonfat milk in PBS-T for 3 h at room temperature (RT) or overnight at 4°C. Primary antibodies were from: Calbiochem 178479 for apoE, Signet 9080 for apoJ, Abcam ab18180 for ABCA1, R&D Systems AF2255 for LDLR, R&D Systems MAB2258 for VLDLR, and Sigma-Aldrich A3853 for actin. Blots were then washed with PBS-T and incubated with HRP-conjugated secondary antibodies at 1:2000 dilution (Amersham Biosciences) for 1 h at RT. Immunoreactive bands were visualized using ECL Plus (Amersham Biosciences) on the Typhoon 9410 (Amersham Biosciences). Data was analyzed using ImageQuant (Molecular Dynamics). Undiluted rat CSF samples (1-2 μl) and 10 μl of 500-fold diluted plasma samples were separated on 4-15% Tris-HCl SDS-PAGE gels and transferred to PVDF membranes. Western blots were carried out as described above. Fresh rat CSF samples (5 μl) were also separated on 4-16% NativePAGE Novex Bis-Tris gel (Invitrogen) and transferred to PVDF membranes. Blots were placed in blocking solution with 5% non-fat milk in phosphate buffer saline with 0.1% Tween-20 (0.1% PBS-T) for 1 h, followed by incubation with primary antibodies to apoE (Calbiochem 178479) and to albumin (Sigma-Aldrich) with 5% nonfat milk in 0.1% PBS-T for 1 h at RT. Blots were washed with 0.1% PBS-T and incubated with HRP-conjugated secondary antibodies at 1:2000 dilution for 1 h at RT. Immunoreactive bands were visualized using SuperSignal West Femto Substrate (34095, Pierce) on the VersaDoc Imaging System (BioRad). Data was analyzed using QuantityOne Analysis Software (BioRad).

### Aβ ELISA

Rat forebrain samples including the cortex and hippocampus from the left hemisphere were homogenized in 4X volume of 50 mM NaCl buffer containing 0.2% diethylamine (DEA) and complete protease inhibitor cocktail (Roche). After homogenization, 1 ml of each sample was boiled at 100°C for 15 min and spun at 14,000 rpm for 30 minutes at 4°C. The supernatant was neutralized to pH 7.4 with 10% volume of 0.5 M Tris-HCL pH 6.8. Black polystyrene plates (3925, Corning) were coated overnight with 2 μg/ml of rodent *N*-terminal polyclonal antibody (Sig-39153, Covance) in 50 mM carbonate-bicarbonate buffer pH 9.4 (C3041, Sigma). Plates were washed in PBS-T and then blocked with 0.1% Tween 20-Superblock in TBS (37545, Pierce) at RT for 3 h. For CSF Aβ42 detection, 50 μl of undiluted samples were added per well in duplicate and for CSF Aβ40, 50 μl of 5-fold diluted samples were added per well in triplicate. For DEA-soluble brain extracts, 50 μl of undiluted sample were added per well in quadruplicate, followed by 50 μl of Aβ40 G2-10 or Aβ42 12F4 alkaline phosphatase-conjugated antibody. After overnight incubation at 4°C, plates were washed and developed using alkaline phosphatase CDP-star substrate (Applied Biosystems). Luminescence counts were measured using the LJL Analyst (Molecular Devices).

### Cholesterol Measurement

Cholesterol measurement was performed using the Amplex Red cholesterol kit according to manufacturing instructions (A12216, Invitrogen). Briefly, the cholesterol standard was prepared using a 5.17 mM stock solution of cholesterol in 1X reaction buffer. 50 μl of 20-fold diluted CSF or 50 μl of 10-fold diluted DEA brain extracts were added per well in triplicate followed by 50 μl of the complete reaction mixture containing 150 mM Amplex Red, 1 U/ml HRP, 1 U/ml cholesterol oxidase, and 0.1 U/ml cholesterol esterase. The reaction mixture was incubated at 37°C for 30 minutes and fluorescence intensity was measured using a fluorescence microplate reader (Molecular Devices).

### In Situ Hybridization

Mouse ABCA1 cDNA was obtained from Open Biosystems (Clone ID 5388972) and mouse apoE cDNA was obtained from Invitrogen (5136415). The plasmids were linearized with EcoRV and PvuII respectively and used to generate antisense ^35^S-UTP labeled cRNA probes (1015 bp and 785 bp) for *in situ *hybridization [58]. Briefly, sagittal 20 μm slide-mounted brain sections were fixed in 4% paraformaldehyde, acetylated with acetic anhydride in triethanolamine and dehydrated. Slides were then hybridized overnight in a sealed humidification chamber with the antisense riboprobe for ABCA1 or apoE mRNA, stringently washed with decreasing concentrations of SSC and treated with RNase A to remove non-specific label. After dehydration, slides were opposed to BioMax MR x-ray film (Kodak) for 18 hours (ABCA1) or 4 hours (apoE), dipped in NTB-2 nuclear emulsion (Kodak) and stored in desiccated lightproof boxes for 3 weeks (ABCA1) or 1 week (apoE) at 4°C. Slides were then developed, counterstained with hematoxylin, and cover-slipped. Film autoradiographs were used to assess the regional distribution of apoE and ABCA1 mRNA expression in the mouse brain. Densitometric images were acquired using the MCID image analysis system (Imaging Research Inc) and Adobe Photoshop was used to extract images from background. A digital camera (Nikon DSM1200C) and a slide scanning system (Aperio ScanScope XT) were used in conjunction with bright-field microscopy to take images of emulsion-coated slides for evaluation of the cellular localization of silver grains.

### Cholesterol efflux assay

Human embryonic kidney (HEK) 293 cells were transiently transfected with apoE3 cDNA or empty vector control using the Lipofectamine reagent (Invitrogen). After overnight incubation, cells were rinsed three times with pre-warmed serum-free media and incubated for 48 h in serum-free media. Conditioned media was concentrated using 10,000 MW cut-out Centricons (Amicon) and protein was measured using the BioRad protein assay reagent. ApoE concentration in the media was estimated by Western blotting against a standard curve of recombinant human apoE3. CCF-STTG1 human astrocytoma cells were plated in 96-well plates and incubated with media containing 12.5 μM of cholesterol for 24 h. Cell were then rinsed three times with pre-warmed serum-free media and treated with serum-free media containing approximately 50 μg apoE/ml or the equivalent total protein amount of concentrated media from empty vector-transfected HEK cells. Following addition of 5 μM T1317 or vehicle (0.05% DMSO), cells were incubated at 37°C for 48 h. Media was then collected and analyzed for total cholesterol (AmplexRed cholesterol kit, Invitrogen) and protein content (BioRad). For ABCA1 knock-down experiments, CCF-STTG1 cells were transfected by eletrophoration (Amaxa) with siRNA to ABCA1 or non-targeting siRNA control (Dharmacon) prior to cholesterol loading and addition of apoE plus 5 μM T1317 as described above. Cell monolayers were lysed with ice-cold PBS containing 1% Triton X-100 and the protease inhibitors Complete (Roche) and PMSF. Equal amounts of total lysate protein were separated on SDS-PAGE and blotted with antibodies to ABCA1 (Abcam) and actin (Sigma).

## Competing interests

SS, SAV, ML, LMC, JJR, and CVZ are full-time employees of Merck and Co., Inc and hold Merck stock options.

## Authors' contributions

SS performed experiments (WB), analyzed data and draft the manuscript; JZ performed experiments (WB, ELISA) and analyzed data; SAV performed experiments (ISH) and analyzed data; NA performed experiments (cholesterol efflux assay); ML performed experiments (ISH); LMC participated in tissue collection; JJR edited the manuscript; CVZ designed and conceived the study, oversaw experiments, analyzed data, and edited the manuscript. All authors have read and approved the final manuscript.
